# Pre-Surgical Endoscopic Biopsies Are Representative of Esophageal and Esophago-Gastric Junction Adenocarcinoma Histologic Classes and Survival Risk

**DOI:** 10.3390/cancers16234045

**Published:** 2024-12-02

**Authors:** Alessandro Gambella, Roberto Fiocca, Marialuisa Lugaresi, Antonietta D’Errico, Deborah Malvi, Paola Spaggiari, Anna Tomezzoli, Luca Albarello, Ari Ristimäki, Luca Bottiglieri, Elena Bonora, Kausilia K. Krishnadath, Gian Domenico Raulli, Riccardo Rosati, Uberto Fumagalli Romario, Giovanni De Manzoni, Jari Räsänen, Sandro Mattioli, Federica Grillo, Luca Mastracci

**Affiliations:** 1 Pathology Unit, Department of Surgical Sciences and Integrated Diagnostics (DISC), University of Genoa, 16132 Genoa, Italy; roberto.fiocca@unige.it (R.F.); federica.grillo@unige.it (F.G.); luca.mastracci@unige.it (L.M.); 2 Department of Medical and Surgical Sciences (DIMEC), Alma Mater Studiorum, University of Bologna, 40126 Bologna, Italy; marialuisa.lugaresi2@unibo.it (M.L.); elena.bonora6@unibo.it (E.B.); sandro.mattioli@unibo.it (S.M.); 3 Division of Thoracic Surgery, Maria Cecilia Hospital, GVM Care & Research Group, Cotignola, 48022 Ravenna, Italy; 4 Pathology Unit, IRCCS Azienda Ospedaliero Universitaria di Bologna, 40138 Bologna, Italy; antonietta.derrico@unibo.it (A.D.); deborah.malvi@aosp.bo.it (D.M.); 5 Department of Experimental, Diagnostic and Specialty Medicine (DIMES), Alma Mater Studiorum, University of Bologna, 40126 Bologna, Italy; 6 Unit of Anatomic Pathology, Humanitas University, 20089 Milan, Italy; paola.spaggiari@humanitas.it; 7 Unit of Anatomic Pathology, Azienda Ospedaliera di Verona, 37122 Verona, Italy; anna.tomezzoli@aovr.veneto.it; 8 Pathology Unit, IRCCS San Raffaele Scientific Institute, 20132 Milan, Italy; albarello.luca@hsr.it; 9 Department of Pathology, HUSLAB and HUS Diagnostic Center, University of Helsinki, 00170 Helsinki, Finland; ari.ristimaki@hus.fi; 10 Helsinki University Hospital, 00170 Helsinki, Finland; 11 Unit of Anatomic Pathology, Istituto Europeo di Oncologia, 20122 Milan, Italy; luca.bottiglieri@ieo.it; 12 IRCCS Azienda Ospedaliero Universitaria di Bologna, 40126 Bologna, Italy; 13 Laboratory of Experimental Medicine and Pediatrics (LEMP), Department of Gastroenterology and Hepatology, University Hospital Antwerp, 2650 Antwerp, Belgium; sheila.krishnadath@uza.be; 14 Pathology Unit, AUSL Area Vasta Romagna, 48100 Ravenna, Italy; g.raulli@ausl.ra.it; 15 Department of Gastrointestinal Surgery, IRCCS San Raffaele Scientific Institute, Vita Salute San Raffaele University, 20132 Milan, Italy; rosati.riccardo@hsr.it; 16 Digestive Surgery, European Institute of Oncology—IRCCS, 20122 Milan, Italy; ubeto.fumagalliromario@ieo.it; 17 Department of Surgery, General and Upper G.I. Surgery Division, University of Verona, 37126 Verona, Italy; giovanni.demanzoni@univr.it; 18 Department of General Thoracic and Esophageal Surgery, Helsinki University Hospital, Helsinki University, 00170 Helsinki, Finland; jari.rasanen@hus.fi; 19 IRCCS San Martino Policlinic Hospital of Genoa, 16132 Genoa, Italy

**Keywords:** esophageal cancer, esophago-gastric junction cancer, diagnostic biopsy, histologic classification, survival risk

## Abstract

**Background and Objectives:** The Esophageal Adenocarcinoma Study Group Europe (EACSGE) recently proposed a granular histologic classification of esophageal–esophago-gastric junctional adenocarcinomas (EA-EGJAs) based on the study of naïve surgically resected specimens that, when combined with the pTNM stage, is an efficient indicator of prognosis, molecular events, and response to treatment. In this study, we compared histologic classes of endoscopic biopsies taken before surgical resection with those of the surgical specimen, to evaluate the potential of the EACSGE classification at the initial diagnostic workup. **Methods:** A total of 106 EA-EGJA cases with available endoscopic biopsies and matched surgical resection specimens were retrieved from five Italian institutions. Histologic classification was performed on all specimens to identify well-differentiated glandular adenocarcinoma (WD-GAC), poorly differentiated glandular adenocarcinoma (PD-GAC), mucinous muconodular carcinoma (MMC), infiltrative mucinous carcinoma (IMC), diffuse desmoplastic carcinoma, diffuse anaplastic carcinoma (DAC), and mixed subtypes. Related risk subgroups (low-risk versus high-risk) were also assessed. The correlations of histologic classes and risk subgroups between diagnostic biopsies and surgical resection specimens were explored with Spearman’s correlation test. Sensitivity, specificity, accuracy, positive predictive value, negative predictive value, true positives, true negatives, false positives, and false negatives were also calculated. **Results:** A strong positive correlation between biopsies and surgical specimens occurred for both histologic classes (coefficient: 0.75, *p* < 0.001) and risk subgroups (coefficient: 0.65, *p* < 0.001). The highest sensitivities and specificities were observed for MMC, IMC, and DAC (100% and 99% for all), followed by WD-GAC (sensitivity 91%, specificity 79%) and PD-GAC (sensitivity 722%, specificity 86%). The low-risk and high-risk groups presented a sensitivity and specificity of 89% and 76% (low-risk) and 76% and 89% (high-risk). **Conclusions:** The EACSGE histologic classification of EA-EGJAs and associated prognostic subgroups can be reliably assessed on pre-operative diagnostic biopsies. Further studies on larger and more representative cohorts of EA-EGJAs will allow us to validate our findings and confirm if the EA-EGJA biopsy histomorphology and clinical TNM staging will be as efficient as the surgical specimen histomorphology and pTNM in predicting patient prognoses and tailoring personalized therapeutic approaches.

## Simple Summary

The surgical specimen histology of esophageal and esophago-gastric junction adenocarcinomas (EA-EGJAs) classified according to the Esophageal Adenocarcinoma Study Group Europe (EACSGE) proposal, eventually combined with the pTNM stage, is an efficient indicator of prognosis, molecular events, and response to treatment. To explore if this histologic classification may be applied to endoscopic biopsies collected at the initial diagnostic workup, we compared the histology of endoscopic and matched surgical specimen tissues collected from 106 cases of EA-EGJA with no neoadjuvant therapy. Histologic classes of endoscopic biopsies and surgical specimen were coincident. Further studies will indicate if EA-EGJA biopsy provides detailed morphological/biological information “per se” for planning therapy, if biopsy histomorphology/clinical TNM crossing is as efficient as the surgical specimen histomorphology/pTNM one, to predict prognosis and to tailor therapy.

## 1. Introduction

Esophageal cancer is the eighth most common cancer and ranks as the sixth leading cause of cancer-related mortality, with approximately 604,000 new cases and 544,000 deaths in 2020 [1,2]. In this scenario, esophageal–esophago-gastric junctional adenocarcinomas (EA-EGJAs) represent a significant clinical challenge, especially in Western countries, where their incidence continuously increase [1,2,3]. In the United States and Northern Europe, EA-EGJAs represent about 60% of new esophageal cancer cases diagnosed [4,5]. In particular, the incidence of EA-EGJAs has increased by nearly eightfold in Western populations since the 1970s, nowadays reaching rates as high as 9.4 per 100,000 men in the United Kingdom [6]. The increase in EA-EGJA incidence contrasts with declining esophageal squamous cell carcinoma (ESCC) rates, highlighting a unique epidemiological trend [5,7,8]. Indeed, while ESCC remains the dominant subtype globally due to its prevalence in regions such as Asia and Africa, EA-EGJAs have emerged as the predominant histological subtype in North America, Europe, and Australia [4,5,8]. This divergence highlights the role of geographic, environmental, and lifestyle factors in disease etiology [9,10,11,12,13,14]. In particular, the rise of EA-EGJAs is closely linked to Barret’s esophagus (BE), which develops due to chronic gastroesophageal reflux disease (GERD) and is characterized by intestinal metaplasia of the esophageal epithelium [15]. Although only a small fraction of patients with BE progress to EA-EGJAs, this condition represents a key target for surveillance and early intervention efforts [6,16].

The socio-economic burden of EA-EGJAs is profound, with substantial implications for healthcare systems worldwide. The economic impact originates not only from the direct costs of diagnosis and treatment—which include histopathological assessment, surgery, chemotherapy, radiation, and endoscopic interventions—but also from indirect costs such as lost productivity and the psychological toll on patients and families. Indeed, the socio-economic impact of EA-EGJAs extends beyond healthcare costs. Patients often experience significant quality-of-life impairments due to the aggressive nature of the disease and the toxicity of treatment regimens. Additionally, disparities in healthcare access contribute to variations in survival outcomes, with lower survival rates observed in minority and socioeconomically disadvantaged populations [5,8,17,18]: the financial burden disproportionately affects lower-income groups due to their limited access to early detection/screening programs and advanced treatment options [5,8,17,18]. The classification of tumors based on histologic subtype supports patient clinical management and has prognostic and predictive implications for subsequent therapeutic approaches [19,20,21,22,23,24,25]. A granular histologic classification of esophageal–esophago-gastric junctional adenocarcinomas (EA-EGJAs) has yet to be determined. Most guidelines and studies [26,27,28], including the latest edition of the WHO classification of digestive system tumors [29], do not report a definite classification system but rather differentiation patterns (i.e., tubular, papillary, mucinous, and signet ring cell patterns) pending further evidence of their clinical relevance. This setting thoroughly differs from other gastro-intestinal tumors, such as gastric adenocarcinomas, that present specific subtypes with associated well-defined prognostic potential [30,31,32,33]. Other than the pathological TNM staging [34] and the WHO three-tier grading system [29], no E-EJGAs prognostic system has been validated. In EA-EGJAs, a number of potential driver mutations and structural genomic alterations have been described [35,36,37,38]. This type of cancer is characterized by an overall picture of genomic instability and accumulation of genetic alterations throughout the disease natural history [39,40,41,42]. However, the significant genetic heterogeneity between patients still makes it difficult to define valuable prognostic molecular signatures that drive tumor onset and development. Considering the worldwide increasing incidence in Western countries [5,18,43,44,45,46,47,48,49,50,51] and the overall poor prognosis (five-year overall survival (OS): about 20%) [17,52,53,54,55] of EA-EGJA, improving and anticipating the prognostic stratification of affected patients is a compelling clinical need.

The Esophageal Adenocarcinoma Study Group Europe (EACSGE) recently proposed an innovative histologic classification of EA-EGJA with significative prognostic implications [56]. Harnessing our experience with EA-EGJA [37,57,58], we tested some of the diagnostic criteria currently used for gastric adenocarcinomas and refined diagnostic definitions of both adenocarcinomas with glandular architecture and rare histotypes. Specifically, we defined the following histologic classes: (I) well-differentiated glandular adenocarcinoma (WD-GAC), (II) poorly differentiated glandular adenocarcinoma (PD-GAC), (III) mucinous muconodular carcinoma (MMC), (IV) infiltrative mucinous carcinoma (IMC), (V) diffuse desmoplastic carcinoma (DDC), (VI) diffuse anaplastic carcinoma (DAC), and (VII) mixed subtypes (MXD) [56]. This classification was tested on a multi-institutional cohort of treatment-naïve, surgically resected EA-EGJAs and was proven to have a significant prognostic impact, improving the 5-year cancer-specific survival (CSS) stratification of early (stage II) and advanced (stage IVa) disease.

In this study, we aim to test the feasibility and efficiency of the EACSGE EA-EGJA histologic classification and related risk subgroups on diagnostic endoscopic biopsies prior to surgical resection. To this end, we retrieved and expanded our previous series of EA-EGJAs and evaluated the correlation between the classification of endoscopic biopsies and matched surgical resection specimens.

## 2. Materials and Methods

### 2.1. Cohort Composition and Data Collection

This is a retrospective multicentric study on EA-EGJAs. Overall, 106 cases diagnosed between January 1998 and December 2023 were included and analyzed. All cases were selected to have both a pre-surgical diagnostic biopsy and the matched surgical specimens. Of these, 75 with available pre-surgical diagnostic biopsies were selected from a previously described series [56] and collected thanks to a joint effort of five institutions belonging to the Esophageal Adenocarcinoma Study Group Europe (EACSGE). Thirty-one additional cases of EA-EGJAs were collected from the Pathology Unit Archives of the IRCCS San Martino Policlinic Hospital-University of Genoa. Cases that underwent neoadjuvant therapy were excluded to prevent possible post-treatment effects on tumor morphology. No specific study protocol was developed for the collection of upper gastro-intestinal tract endoscopy procedures and biopsies, but rather, a scientific societies recommendations-based approach was used by each institution. The detailed inclusion and exclusion criteria are reported in Supplementary Methods.

All samples were anonymized by a member of our group not directly involved in this study. All procedures were performed in accordance with the ethical standards of the Human Experimentation Institutional Review Board (IRB) of the IRCCS San Martino Policlinic Hospital-University of Genoa (IRB approval number: 101/2021) and of the IRST IRCCS Area Vasta Romagna CEIIAV-Italy (IRB approval number: L3P1223-109/2016-7353/51/2016) and in accordance with the World Medical Association Declaration of Helsinki of 1964 and later versions.

### 2.2. Histologic Classification and Variable Evaluation

To prevent evaluation biases, all samples (i.e., biopsy and matched surgical specimens) of the same patient were anonymized, separated, and assessed independently by a pathologist with dedicated training and expertise on EA-EGJAs (L.M.).

All cases were reviewed and reclassified using the EACSGE histologic classification [56] reported in Table 1.

**Figure 1:**
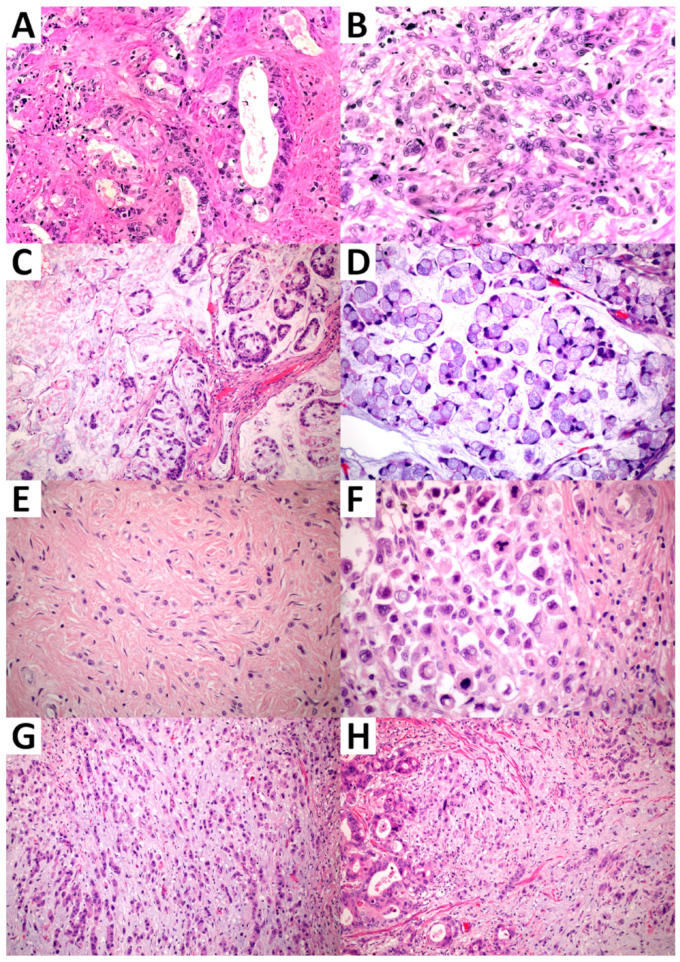
Representative images of EA-EGJA histologic classes. (**A**) Well-differentiated glandular adenocarcinoma (WD-GAC) showing well-formed glands (10× original magnification); (**B**) poorly differentiated glandular adenocarcinoma (PD-GAC) showing loss of glandular structure but preserved cell cohesion; scant glandular structures can still be recognized (10× original magnification); (**C**) mucinous muconodular carcinoma (MMC) showing mucin lakes with floating glandular structure and cluster of cohesive cells (5× original magnification); (**D**) infiltrative mucinous carcinoma (IMC) with poorly cohesive tumor cells, isolated or in small aggregates, showing signet ring cell features floating in mucin (10× original magnification); (**E**) diffuse desmoplastic carcinoma (DDC) showing marked desmoplasia with scant, poorly cohesive isolated cells or in small aggregates (10× original magnification); (**F**) diffuse anaplastic carcinoma (DAC) characterized by poorly cohesive and highly atypical tumor cells (10× original magnification); (**G**,**H**) mixed subtype (MXD) showing two or more distinct histologic components (glandular/tubular/papillary and poorly cohesive/signet ring) (5× original magnification).

Representative images of EA-EGJAs and related histologic EACSGE classes are depicted in Figure 1.

Based on the histologic classification, all cases were stratified as low- or high-risk according to the criteria previously reported by the EACSGE [56]. Briefly, the WD-GAC, MMC, and DDC histologic classes characterized the low-risk group and identified patients with improved cancer-specific survival (CSS), whereas the PD-GAC, IMC, DAC, and MIX classes characterized high-risk patients with worse prognoses [56].

The following additional features were also considered and assessed: comprehensive number of biopsy samples per patient, number of biopsies with invasive neoplasia (samples with non-neoplastic mucosa, non-invasive neoplasia, or necro-inflammatory materials were excluded), histologic classification according to Lauren’s type (intestinal versus diffuse versus unclassified morphology) [59], pattern of growth according to Ming’s classification (expanding versus infiltrative) [60], percentage of glandular structure loss (including poorly differentiated clusters), grade according to WHO Classification of Tumors 2019 (G1 well-formed glands in >95%, G2 in 50–95%, G3 in <50% of tumor) [29], and percentage of signet ring cells [61].

Following the histopathological assessment, all biopsy samples were matched to the related surgical resection specimens for the subsequent correlation analysis.

### 2.3. Statistical Analysis

Statistical analyses were performed by the EACSGE statistic unit using the SPSS15.0 software package (SPSS Inc., Chicago, IL, USA), R Software (version 4.2.2; The R Foundation for Statistical Computing, Vienna, Austria), and RStudio (version 2022.12.0 + 353; RStudio, Boston, MA, USA) as previously reported [56]. Continuous variables were reported as medians and interquartile ranges (IQRs) and categorical variables as numbers and percentages. Spearman’s correlation test was used to explore the correlation between histopathological subclasses of diagnostic biopsies and surgical resection specimens, and their related risk class (low- and high-risk). Histologic classes were analyzed for sensitivity, specificity, accuracy, positive predictive value, negative predictive value, and true positive, true negative, false positive, and false negative status. For this analysis, the surgical resection specimens were used as the reference gold standard due to the more representative nature of this sample compared to the diagnostic biopsy. ROC analysis was used to identify the minimum number of biopsies to allow a specific and correct histologic classification with the best sensitivity and specificity metrics. A *p*-value < 0.05 was considered significant.

## 3. Results

### 3.1. Baseline Characteristics: Our Cohort Is Representative of the EA-EGJA Population

This study is based on a multicentric series of 106 EA-EGJA cases. To ensure the validity and reproducibility of our data, we first evaluated whether the demographic, clinical, and pathological features of our cases were in line with the current literature.

Our series presented a predominance of male patients (84/106, 79.2%) and a median age of 69.5 years (IQR: 59–76). We observed that most cases were Type II (54/106, 50.9%) according to the Siewert classification [62,63] and presented disease-free margins (R0) following surgical resection (98/106, 92.4%). Regarding the pathological features, most cases presented the pT3 stage (73/106, 68.9%), nodal metastases (81/106, 76%), and an absence of distant metastases (93/101, 92.1%), resulting in stage IIIb being the most frequent pathological stage (45/106, 42.4%). The median follow-up of our cohort was 15 months (IQR: 6–36). At the end of the follow-up period, 39 patients (51.3%) presented disease recurrence, and 43 (57.3%) were deceased. Detailed clinicopathological data of our series are reported in Table S1.

Overall, our cohort aligned with the available EA-EGJA evidence, including male prevalence, median age, histopathological features, and survival data [52,64,65].

### 3.2. Histologic Classes of EA-EGJA: Data from Diagnostic Biopsies and Surgical Resection Specimens

Reassured that our series was representative of the E-EGAJ population, we delved into the histologic analysis.

Definitions of EA-EGJA histologic subtypes and rare variants are poorly defined in the literature. To provide a granular classification, we decided to adapt the (more detailed) criteria available for gastric adenocarcinomas [31,61], using the EACSGE classification developed on surgical specimens [56]. Specifically, we classified all samples (i.e., both biopsies and surgical specimens) as well-differentiated glandular adenocarcinoma (WD-GAC), poorly differentiated glandular adenocarcinoma (PD-GAC), mucinous muconodular carcinoma (MMC), invasive muconodular carcinoma (IMC), diffuse desmoplastic carcinoma (DDC), diffuse anaplastic carcinoma (DAC), and mixed adenocarcinoma (MIX). Of note, we decided to first analyze biopsies and surgical resection specimens independently (i.e., without knowledge of related matching).

We started by classifying the diagnostic biopsies and observed that most cases were WD-GAC (52/106, 49.1%) or PD-GAC (40/106, 37.7%), as expected. Rare variants (MMC, IMC, DDC, and DAC) and mixed forms (MIX) were marginally represented (4/106, 5/106, 0/106, 5/106, and 0/106, respectively). Analysis of the surgical specimen classification provided slightly different results. We still classified most cases as WD-GAC (43/106, 40.6%) and PD-GAC (43/106, 40.6%). However, we observed a more heterogeneous scenario in the remaining cases: all rare variants were represented; specifically, we identified three MMCs, four IMCs, one DDCs, and three DACs (3/106). In addition, we classified nine cases as mixed (MIX), of which most were PD-GAC combined with DAC (five cases). Details of the composition of MIX cases are reported in Table S2.

Based on the histologic classes and following the approach we previously described [56], we then stratified the cases as low (WD-GAC, MMC, and DDC) or high (PD-GAC, IMC, DAC, and MIX) prognostic risk. Of note, the distributions of low- and high-risk cases in diagnostic biopsies and surgical resection specimens were similar: 52.8% of biopsies (56/106) resulted in the low-risk category compared to 44.3% (47/106) of the surgical specimen cohort.

Detailed results of the histologic classification and related risk subgroups of both biopsies and surgical resection specimens are reported in Table 2.

Overall, the biopsies and surgical resection specimens showed comparable distribution of samples in histologic classes and related survival risk subgroups. As expected, rare and mixed classes were partially identified on pre-surgical biopsies.

### 3.3. Biopsies Provided Reliable Histologic Classification and Survival Risk Subgroup Assessment

Considering the comparable distribution—with few exceptions—between the biopsies and surgical specimens, we were interested in assessing which histologic classes caused the discrepancy. To this end, we matched the biopsies to the related surgical specimens and compared the histologic classes and related risk groups.

First, we started with the histologic classes and observed a strong, positive monotonic correlation between the biopsies and surgical specimen classification (Spearman’s rho correlation coefficient: 0.75, *p* < 0.001). Based on this result, we further evaluated the sensitivity, specificity, accuracy, positive predictive value (PPV), negative predictive value (NPV), true positives (TPs), false positives (FPs), true negatives (TNs), and false negatives (FNs) of the histologic classes defined on the biopsies using the results of the surgical specimens as reference. This analysis was available only for the WD-GAC, PD-GAC, MMC, IMC, and DAC subclasses, as no biopsies were classified as DDC and MIX. Overall, we observed good performance: the highest sensitivity and specificity were observed for the MMC, IMC, and DAC subclasses (100% and 99% for all of them, respectively), followed by WD-GAC (sensitivity 91%, specificity 79%) and PD-GAC (sensitivity 72%, specificity 86%). Detailed results of all metrics are reported in Table 3.

**Figure 2:**
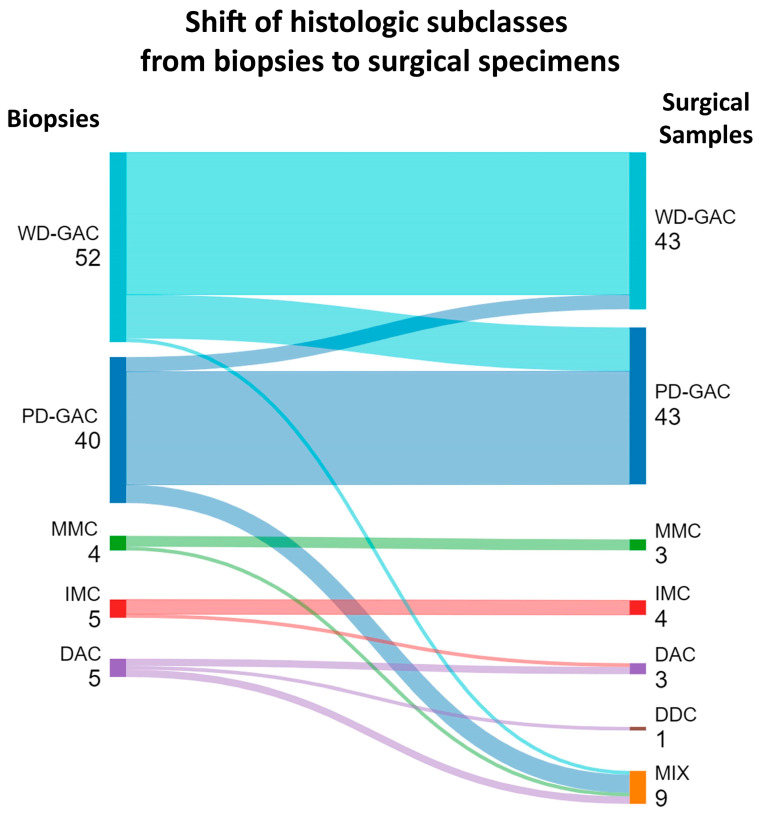
Sankey diagram highlighting the shift of histologic class from biopsies to surgical specimens. DAC: diffuse anaplastic carcinoma; DDC: diffuse desmoplastic carcinoma; IMC: invasive muconodular carcinoma; MIX: mixed adenocarcinoma; MMC: mucinous muconodular carcinoma; PD-GAC: poorly differentiated glandular adenocarcinoma; WD-GAC: well-differentiated glandular adenocarcinoma.

Following this analysis, we decided to further detail the misdiagnosed classes. Regarding WD-GAC, most cases (39/43) were correctly defined, but four were initially classified as PD-GAC on biopsy. Similarly, 31 of the 43 PD-GAC cases were identified on biopsy, but 12 were instead classified as WD-GAC. Regarding the rare variants, all MMC (3/3) and IMC (4/4) cases corresponded between the biopsies and surgical samples. Unlike the other two classes, none of the MIX (0/9) and DDC (0/1) cases were initially identified on biopsy. Considering the heterogeneous nature of the MIX cases, we decided to evaluate whether the mismatch was related to a sampling issue (i.e., biopsy showing only one of the MIX components). Indeed, we observed that this happened for all the MIX cases. Specifically, seven MIX cases that presented PD-GAC and DAC components were classified as PD-GAC (5/9) and DAC (2/9), one MIX case with WD-GAC and DAC components was classified as WD-GAC, and one MIX case with MMC and DAC components was classified as MMC. A graphical representation of the histologic class correlation between the biopsies and surgical samples is shown in Figure 2.

**Figure 3:**
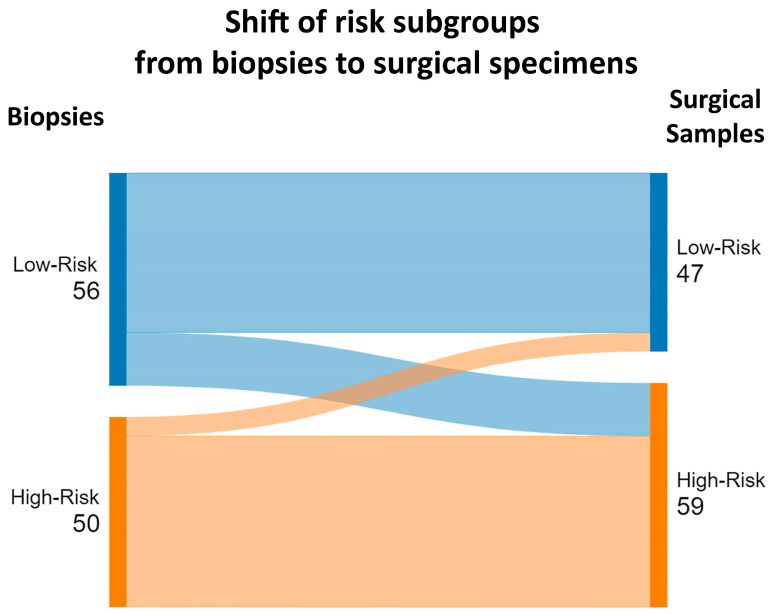
Sankey diagram highlighting the shift of survival risk subgroups between the biopsies and surgical specimens.

We performed the same analysis for the histologic-derived prognostic risk groups (low-risk versus high-risk). Similar to the histologic classification analysis, we observed a strong, positive monotonic correlation between the biopsies and surgical specimen risk groups (Spearman’s rho correlation coefficient: 0.65, *p* < 0.001). We also calculated sensitivity, specificity, accuracy, PPV, NPV, TPs, FPs, FNs, and TNs. The low-risk and high-risk groups presented sensitivities and specificities of 89% and 76%, and 76% and 89%, respectively. In total, 42 of the 47 low-risk cases and 45 of the 59 high-risk cases were identified on biopsies. Detailed metrics and a graphical representation are presented in Table 4 and Figure 3.

These results showed some variability between the biopsies and surgical specimen histologic classes, which is thoroughly mitigated when considering survival risk subgroups.

### 3.4. Numbers Matter: How Many Biopsies Do We Need to Classify EA-EGJAs?

Considering the relevance of pre-surgical diagnostic biopsies in EA-EGJA patient clinical management, we decided to explore their metrics further. In particular, we collected the overall number of biopsies available for assessment per patient and the specific number of biopsies positive for invasive adenocarcinoma per patient.

**Figure 4:**
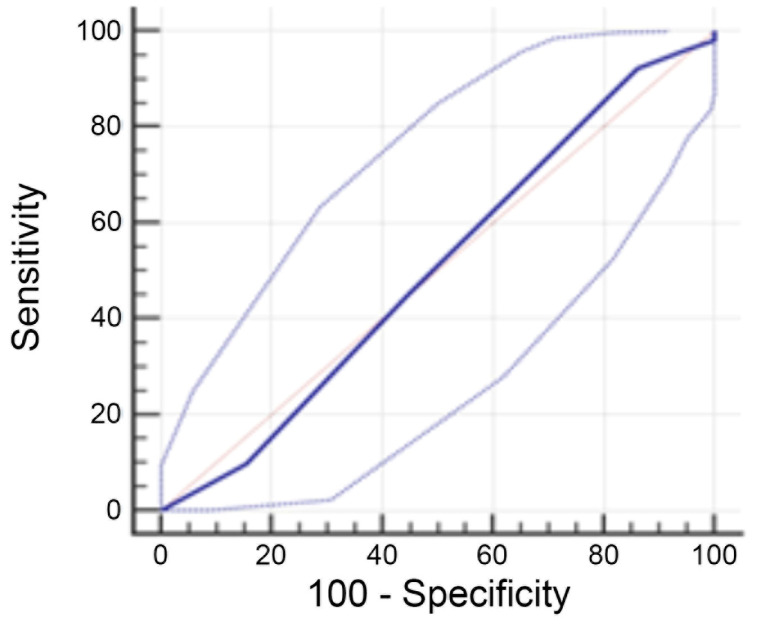
ROC curve for adequate histologic classification based on the number of biopsies with invasive EA-EGJA. Orange line: reference line (line of no-discrimination with an AUC = 0.5); solid dark blue line: ROC curve for 5 biopsies (AUC = 0.503).

Our series had a median of four biopsies per case (IQR: 3–6), whereas the median number of biopsies with evident features of invasive EA-EGJA was three per case (IQR: 2–4). We then performed an ROC curve analysis using the Youden index method and observed that the optimal cutoff point of biopsy number with invasive EA-EGJA required to classify EA-EGJA appropriately was five (95%CI: 4–7; sensitivity: 92.2%, specificity: 13.8%), but the AUC was low at 0.503 (standard error: 0.05; 95%CI: 0.41–0.60; Figure 4).

Despite representing a trend, these results suggested that improved agreement between biopsies and surgical resected specimens can be achieved if at least five biopsies representative of EA-EGJA are available for histologic assessment.

## 4. Discussion

In this study, we demonstrated that pre-surgical diagnostic biopsies of EA-EGJA can provide reliable histologic classification and related risk stratification. As part of an EACSGE initiative, we collected and analyzed 106 EA-EGJA biopsies and matched surgical specimens. Both histologic classification and related survival risk stratification evaluated on biopsies were significantly related to the corresponding assessment on surgical resection specimens (correlation index: 0.754 and 0.653, respectively; *p* < 0.001, both). The sensitivity and specificity of biopsy-based classification were in line with these data, ranging between 72% and 100% (sensitivity) and 79% and 99% (specificity) depending on the specific class considered. These results support the implementation of histologic classification and risk group stratification on EA-EGJA biopsies in daily diagnostic practice.

In our cohort, most cases were well-differentiated and poorly differentiated glandular carcinomas (WD-GAC and PD-GAC, respectively). Focusing on these two classes, biopsies allowed for an accurate histotype classification in 81.1% of the cases (86/106). A non-negligible proportion of the cases (12/43, 27.9%) were “downgraded” from WD-GAC on biopsy to PD-GAC on the surgical specimen. This discrepancy is likely due to the sampling bias inherent to endoscopy biopsy, which typically collects the superficial and often more differentiated portion of the tumor. In contrast, the deep infiltrating (and often dedifferentiated) tumor components can be under-represented or entirely missed with biopsy sampling. A similar consideration emerged from the analysis of the mixed class (MIX). In our cohort, nine cases were MIX (8.5%) on surgical resection specimens, but none of these were identified on biopsies. Once we matched the biopsy back to the specific surgical resection specimen, we observed that the apparent mismatch was entirely related to a sampling issue, as the biopsies presented only one of the two histotypes eventually composing the MIX case on the surgical resection specimen. These findings and considerations emphasize the importance of correlating endoscopic findings with histopathological features and, in particular, highlight the need for adequate biopsy sample collection. To this end, our data suggest that a higher number of biopsies would improve the accuracy of histologic classification and risk categorization of EA-EGJAs. Although statistical significance was not reached, a number of biopsy samples greater than five offered the best sensitivity (albeit with low specificity) in correctly subclassifying EA-EGJAs. This finding is in line with data reported in other contexts, including the number of neoplastic biopsies representative of gastric adenocarcinoma required for accurate HER-2 assessment [57].

Despite the inherent limitations of biopsy samples, it is worth mentioning that the correct survival risk category was assigned in most cases. In our previous work, histology-based risk subgroups improved patient survival stratification across all EA-EGJA pathological stages [56]. Recent advances in high-throughput genomic and transcriptomic profiling have identified several molecular subtypes of EA-EGJAs with prognostic potential [42,58,66,67,68,69]. While fascinating and innovative, the data collected from these assays are generally burdened by well-known limitations, including the limited availability worldwide, the prolonged turnaround time, and the associated substantial cost. With regards to patients who are diagnosed with advanced non-operable tumors, biopsy tissue is often the only tissue available and is therefore extremely precious. The EACSGE classification and related risk stratification can be easily, and reliably, applied on such tissue without the need for tissue use (and possible wastage). Furthermore, we would like to stress that our EACSGE classification does not aim to replace other approaches but rather integrate with them by providing a prompt and informative tool that can be easily integrated into the daily diagnostic routine.

Other studies in the literature focused on the histopathological features of EA-EGJA tissue biopsy samples, especially in terms of histopathological changes between the biopsy samples and subsequent larger specimens [70]. Jiang et al. analyzed 68 patients undergoing endoscopic submucosal dissection (ESD) for early EA-EGJAs and compared pre-operative biopsy findings with post-ESD specimens [70]. About 70% of cases displayed a diagnosis change, with all showing pathological upstaging to more advanced stages. Similarly to our study, the authors observed and highlighted the limitations of endoscopic biopsy in accurately assessing the pathological status of tumors, especially for deeper infiltrative stages. The limited representation of rare variants partially hinders the impact of our findings, which will benefit from further validation on larger cohorts. Furthermore, most EA-EGJAs are now treated pre-operatively with chemoradiation and, therefore, will show histologic changes consistent with treatment effects on surgical resection specimens. Consequently, our classification can be implemented only on neoadjuvant-naïve samples, such as pre-treatment biopsies or (rarely performed) mucosal/submucosal resection specimens, representing a minor yet potentially relevant part of cases. Furthermore, the use of narrow-band imaging (NBI) with magnifying endoscopy might increase endoscopic sampling sensitivity and specificity and should be further tested in dedicated studies [71,72,73,74].

Based on our evidence and considerations, it would be interesting to further address the role and impact of the endoscopy procedure per se, especially in terms of the biopsy collection procedure, on the histopathologic diagnosis and overall patient clinical management.

## 5. Conclusions

In conclusion, we demonstrated that the histologic EACSGE classification of EA-EGJAs and associated prognostic subgroups can be reliably assessed on pre-operative diagnostic biopsies. Pending further validation on larger and more representative cohorts of EA-EGJAs, this classification can improve tumor granular classification and patients’ early prognostic stratification. We envision that further studies will validate present findings and indicate if the EA/EGJAs biopsy histomorphology/clinical TNM crossing will be as efficient as the surgical specimen histomorphology/pTNM one in predicting prognoses and tailoring therapy.

## Figures and Tables

**Table 1:** Histologic EACSGE classification and related definitions used in this study.

Class	Defining Criteria
Well-Differentiated Glandular Adenocarcinoma (WD-GAC)	Carcinomas showing well-defined glandular formation, a pattern of growth that is mostly expansive, and a loss of glandular architecture in ≤5% of the tumor area
Poorly Differentiated Glandular Adenocarcinoma (PD-GAC)	Similar to WD-GAC, but the loss of glandular architecture is observed in >5% of the tumor area; there is a loss of cell cohesion (i.e., clusters of individual cells are observed); and the pattern of growth is mostly invasive
Mucinous Muconodular Carcinoma (MMC)	Carcinomas showing mucinous component (i.e., mucin lakes with floating tumor cells, a predominance of extracellular mucin over tumor cells) in ≥80% of the tumor; moderate cellular anaplasia; and a mostly expansive growth pattern
Invasive Mucinous Carcinoma (IMC)	Similar to MMC, but the pattern of growth is mostly infiltrative; higher cellularity is observed; and tumor cells show more pronounced cellular atypia
Diffuse Desmoplastic Carcinoma (DDC)	Non-cohesive carcinomas characterized by a fibroblast-rich desmoplastic stroma embedding scant clusters or individual tumor cells with moderate atypia
Diffuse Anaplastic Carcinoma (DAC)	Carcinomas characterized by scarce stroma and are poorly cohesive; large- to medium-sized tumor cells with large, pleomorphic, highly atypical nuclei and prominent nucleoli; tumor cellularity and proliferative rates are high
Mixed (MIX)	Tumors with two (or more) distinct histologic components (glandular/tubular/papillary and poorly cohesive/signet ring) according to the WHO criteria for gastric adenocarcinoma (no specific criteria are available for E-EGJCA)

**Table 2:** Histologic classes and risk stratification of biopsies and surgical resection specimens.

	Biopsies (n = 106)	Surgical Resection Specimens (n = 106)
***Histologic Class***		
**WD-GAC**	52 (49.1%)	43 (40.6%)
**PD-GAC**	40 (37.7%)	43 (40.6%)
**MMC**	4 (3.8%)	3 (2.8%)
**IMC**	5 (4.7%)	4 (3.8%)
**DDC**	0 (0%)	1 (0.9%)
**DAC**	5 (4.7%)	3 (2.8%)
**MIX**	0 (0%)	9 (8.5%)
***Risk Stratification***		
**Low-Risk**	56 (52.8%)	47 (44.3%)
**High-Risk**	50 (47.2%)	59 (55.7%)

**Table 3:** Biopsy performance metrics in defining histologic class.

Histologic Class	Sensitivity	Specificity	Accuracy	PPV	NPV	TP	FP	FN	TN	Total
WD-GAC	91%	79%	84%	75%	93%	39	13	4	50	106
PD-GAC	72%	86%	80%	78%	82%	31	9	12	54	106
MMC	100%	99%	99%	75%	100%	3	1	0	102	106
IMC	100%	99%	99%	80%	100%	4	1	0	101	106
DAC	100%	99%	99%	75%	100%	3	1	0	102	106

DAC: diffuse anaplastic carcinoma; FN: false negative; FP: false positive; IMC: invasive muconodular carcinoma; MMC: mucinous muconodular carcinoma; NPV: negative predictive value; PD-GAC: poorly differentiated glandular adenocarcinoma; PPV: positive predictive value; TN: true negative; TP: true positive; WD-GAC: well-differentiated glandular adenocarcinoma.

**Table 4:** Biopsy performance metrics in defining survival risk groups.

Risk Group	Sensitivity	Specificity	Accuracy	PPV	NPV	TP	FP	FN	TN	Total
Low-risk	89%	76%	82%	75%	90%	42	14	5	45	106
High-risk	76%	89%	82%	90%	75%	45	5	14	42	106

FN: false negative; FP: false positive; NPV: negative predictive value; PPV: positive predictive value; TN: true negative; TP: true positive.

## Data Availability

The data supporting the findings of this study are not publicly available due to privacy or ethical restrictions but can be obtained upon reasonable request from the corresponding author.

## Supplementary Materials

The following supporting information can be downloaded at https://www.mdpi.com/article/10.3390/cancers16234045/s1, Supplementary Methods; Table S1: Clinicopathological features of our series; Table S2: Details of components of cases with mixed histopathologic subclass.

## References

[1] Morgan E., Soerjomataram I., Rumgay H., Coleman H.G., Thrift A.P., Vignat J., Laversanne M., Ferlay J., Arnold M. (2022). The Global Landscape of Esophageal Squamous Cell Carcinoma and Esophageal Adenocarcinoma Incidence and Mortality in 2020 and Projections to 2040: New Estimates From GLOBOCAN 2020. Gastroenterology.

[2] Lander S., Lander E., Gibson M.K. (2023). Esophageal Cancer: Overview, Risk Factors, and Reasons for the Rise. Curr. Gastroenterol. Rep..

[3] Yang H., Wang F., Hallemeier C.L., Lerut T., Fu J. (2024). Oesophageal cancer. Lancet.

[4] Umar S.B., Fleischer D.E. (2008). Esophageal cancer: Epidemiology, pathogenesis and prevention. Nat. Clin. Pract. Gastroenterol. Hepatol..

[5] Thrift A.P. (2021). Global burden and epidemiology of Barrett oesophagus and oesophageal cancer. Nat. Rev. Gastroenterol. Hepatol..

[6] Weaver J.M., Ross-Innes C.S., Fitzgerald R.C. (2014). The ‘-omics’ revolution and oesophageal adenocarcinoma. Nat. Rev. Gastroenterol. Hepatol..

[7] Edgren G., Adami H.O., Weiderpass E., Nyren O. (2013). A global assessment of the oesophageal adenocarcinoma epidemic. Gut.

[8] Arnold M., Ferlay J., van Berge Henegouwen M.I., Soerjomataram I. (2020). Global burden of oesophageal and gastric cancer by histology and subsite in 2018. Gut.

[9] Quante M., Wang T.C., Bass A.J. (2023). Adenocarcinoma of the oesophagus: Is it gastric cancer?. Gut.

[10] Dong J., Thrift A.P. (2017). Alcohol, smoking and risk of oesophago-gastric cancer. Best. Pract. Res. Clin. Gastroenterol..

[11] Mueller J., Werner M., Stolte M. (2004). Barrett’s esophagus: Histopathologic definitions and diagnostic criteria. World J. Surg..

[12] Pohl H., Pech O., Arash H., Stolte M., Manner H., May A., Kraywinkel K., Sonnenberg A., Ell C. (2016). Length of Barrett’s oesophagus and cancer risk: Implications from a large sample of patients with early oesophageal adenocarcinoma. Gut.

[13] Fitzgerald R.C. (2005). Barrett’s oesophagus and oesophageal adenocarcinoma: How does acid interfere with cell proliferation and differentiation?. Gut.

[14] Grillo F., Mastracci L., Saragoni L., Vanoli A., Limarzi F., Gullo I., Ferro J., Paudice M., Parente P., Fassan M. (2020). Neoplastic and pre-neoplastic lesions of the oesophagus and gastro-oesophageal junction. Pathologica.

[15] Anaparthy R., Sharma P. (2014). Progression of Barrett oesophagus: Role of endoscopic and histological predictors. Nat. Rev. Gastroenterol. Hepatol..

[16] Salimian K.J., Birkness-Gartman J., Waters K.M. (2022). The path(ology) from reflux oesophagitis to Barrett oesophagus to oesophageal adenocarcinoma. Pathology.

[17] Rubenstein J.H., Shaheen N.J. (2015). Epidemiology, Diagnosis, and Management of Esophageal Adenocarcinoma. Gastroenterology.

[18] Malhotra G.K., Yanala U., Ravipati A., Follet M., Vijayakumar M., Are C. (2017). Global trends in esophageal cancer. J. Surg. Oncol..

[19] Zhang A., Li Y., Zhang H., Liu H., Han C., Shi G. (2023). Comparison of TNM AJCC/UICC 8th with JES 11th staging systems for prognostic prediction in patients with esophageal squamous cell carcinoma who underwent radical (chemo) radiotherapy in China. J. Cancer Res. Ther..

[20] Cree I.A., Lokuhetty D., Tan P.H. (2022). The World Health Organization Classification of Tumors and External Quality Assurance for Immunohistochemistry and Molecular Pathology. Arch. Pathol. Lab. Med..

[21] Zhang X.Q., Miao C.W., Liu L.P., Wang C.L., Chen J.Z., Li W.H., Hu X.D. (2023). The prognostic value of 11(th) Japanese classification and 8(th) AJCC staging systems in Chinese patients with esophageal squamous cell carcinoma. J. Cardiothorac. Surg..

[22] Zhang D., Zheng Y., Wang Z., Huang Q., Cao X., Wang F., Liu S. (2017). Comparison of the 7th and proposed 8th editions of the AJCC/UICC TNM staging system for esophageal squamous cell carcinoma underwent radical surgery. Eur. J. Surg. Oncol..

[23] Udagawa H., Ueno M. (2018). Comparison of two major staging systems of esophageal cancer-toward more practical common scale for tumor staging. Ann. Transl. Med..

[24] Jiang G., Wang Z., Cheng Z., Wang W., Lu S., Zhang Z., Anene C.A., Khan F., Chen Y., Bailey E. (2024). The integrated molecular and histological analysis defines subtypes of esophageal squamous cell carcinoma. Nat. Commun..

[25] Lin J.P., Chen X.F., Zhou H., Zhuang F.N., He H., Chen W.J., Wang F., Liu S.Y. (2024). The association between histological subtypes and lymph node metastasis and prognosis in early esophageal cancer: A population-based study. Eur. J. Cancer Prev..

[26] Ajani J.A., Barthel J.S., Bentrem D.J., D’Amico T.A., Das P., Denlinger C.S., Fuchs C.S., Gerdes H., Glasgow R.E., Hayman J.A. (2011). Esophageal and esophagogastric junction cancers. J. Natl. Compr. Canc Netw..

[27] Ajani J.A., D’Amico T.A., Bentrem D.J., Cooke D., Corvera C., Das P., Enzinger P.C., Enzler T., Farjah F., Gerdes H. (2023). Esophageal and Esophagogastric Junction Cancers, Version 2.2023, NCCN Clinical Practice Guidelines in Oncology. J. Natl. Compr. Canc Netw..

[28] Khan N., Donohoe C.L., Phillips A.W., Griffin S.M., Reynolds J.V. (2020). Signet ring gastric and esophageal adenocarcinomas: Characteristics and prognostic implications. Dis. Esophagus.

[29] Nagtegaal I.D., Odze R.D., Klimstra D., Paradis V., Rugge M., Schirmacher P., Washington K.M., Carneiro F., Cree I.A. (2020). The 2019 WHO classification of tumours of the digestive system. Histopathology.

[30] Ferrari M., Ghislandi E., Landonio G., Majno M., Porretta T., Scanzi F. (1992). Histology as a prognostic factor in early gastric cancer. Tumori.

[31] Chiaravalli A.M., Klersy C., Vanoli A., Ferretti A., Capella C., Solcia E. (2012). Histotype-based prognostic classification of gastric cancer. World J. Gastroenterol..

[32] Zhu Z., Sun X., Wang J., Sun Z., Wang Z., Zheng X., Xu H. (2014). Histopathology-based prognostic score is independent prognostic factor of gastric carcinoma. BMC Cancer.

[33] Liu K., Wan J., Bei Y., Chen X., Lu M. (2017). Prognostic Impact of Different Histological Types on Gastric Adenocarcinoma: A Surveillance, Epidemiology, and End Results Database Analysis. Pathol. Oncol. Res..

[34] Rice T.W., Patil D.T., Blackstone E.H. (2017). 8th edition AJCC/UICC staging of cancers of the esophagus and esophagogastric junction: Application to clinical practice. Ann. Cardiothorac. Surg..

[35] Secrier M., Li X., de Silva N., Eldridge M.D., Contino G., Bornschein J., MacRae S., Grehan N., O’Donovan M., Miremadi A. (2016). Mutational signatures in esophageal adenocarcinoma define etiologically distinct subgroups with therapeutic relevance. Nat. Genet..

[36] The Cancer Genome Atlas Research Network (2017). Integrated genomic characterization of oesophageal carcinoma. Nature.

[37] Orsini A., Mastracci L., Bozzarelli I., Ferrari A., Isidori F., Fiocca R., Lugaresi M., D’Errico A., Malvi D., Cataldi-Stagetti E. (2023). Correlations between Molecular Alterations, Histopathological Characteristics, and Poor Prognosis in Esophageal Adenocarcinoma. Cancers.

[38] Lim H., Gingras M.C., Zhao J., Byun J., Castro P.D., Tsavachidis S., Hu J., Doddapaneni H., Han Y., Muzny D.M. (2024). Somatic mutations of esophageal adenocarcinoma: A comparison between Black and White patients. Sci. Rep..

[39] Isidori F., Malvi D., Fittipaldi S., Forcato C., Bozzarelli I., Sala C., Raulli G., D’Errico A., Fiorentino M., Seri M. (2018). Genomic profiles of primary and metastatic esophageal adenocarcinoma identified via digital sorting of pure cell populations: Results from a case report. BMC Cancer.

[40] Frankell A.M., Jammula S., Li X., Contino G., Killcoyne S., Abbas S., Perner J., Bower L., Devonshire G., Ococks E. (2019). The landscape of selection in 551 esophageal adenocarcinomas defines genomic biomarkers for the clinic. Nat. Genet..

[41] Ahuja P., Yadav R., Goyal S., Yadav C., Ranga S., Kadian L. (2023). Targeting epigenetic deregulations for the management of esophageal carcinoma: Recent advances and emerging approaches. Cell Biol. Toxicol..

[42] Greenawalt D.M., Duong C., Smyth G.K., Ciavarella M.L., Thompson N.J., Tiang T., Murray W.K., Thomas R.J., Phillips W.A. (2007). Gene expression profiling of esophageal cancer: Comparative analysis of Barrett’s esophagus, adenocarcinoma, and squamous cell carcinoma. Int. J. Cancer.

[43] Pera M., Manterola C., Vidal O., Grande L. (2005). Epidemiology of esophageal adenocarcinoma. J. Surg. Oncol..

[44] Keeney S., Bauer T.L. (2006). Epidemiology of adenocarcinoma of the esophagogastric junction. Surg. Oncol. Clin. N. Am..

[45] Agarwal S., Bell M.G., Dhaliwal L., Codipilly D.C., Dierkhising R.A., Lansing R., Gibbons E.E., Leggett C.L., Kisiel J.B., Iyer P.G. (2024). Population Based Time Trends in the Epidemiology and Mortality of Gastroesophageal Junction and Esophageal Adenocarcinoma. Dig. Dis. Sci..

[46] Smyth E.C., Lagergren J., Fitzgerald R.C., Lordick F., Shah M.A., Lagergren P., Cunningham D. (2017). Oesophageal cancer. Nat. Rev. Dis. Primers.

[47] Sheikh M., Roshandel G., McCormack V., Malekzadeh R. (2023). Current Status and Future Prospects for Esophageal Cancer. Cancers.

[48] Schlansky B., Dimarino A.J., Loren D., Infantolino A., Kowalski T., Cohen S. (2006). A survey of oesophageal cancer: Pathology, stage and clinical presentation. Aliment. Pharmacol. Ther..

[49] Muir C.S., McKinney P.A. (1992). Cancer of the oesophagus: A global overview. Eur. J. Cancer Prev..

[50] Patel N., Benipal B. (2018). Incidence of Esophageal Cancer in the United States from 2001–2015: A United States Cancer Statistics Analysis of 50 States. Cureus.

[51] Pennathur A., Gibson M.K., Jobe B.A., Luketich J.D. (2013). Oesophageal carcinoma. Lancet.

[52] Bray F., Laversanne M., Sung H., Ferlay J., Siegel R.L., Soerjomataram I., Jemal A. (2024). Global cancer statistics 2022: GLOBOCAN estimates of incidence and mortality worldwide for 36 cancers in 185 countries. CA Cancer J. Clin..

[53] He H., Chen N., Hou Y., Wang Z., Zhang Y., Zhang G., Fu J. (2020). Trends in the incidence and survival of patients with esophageal cancer: A SEER database analysis. Thorac. Cancer.

[54] Domper Arnal M.J., Ferrandez Arenas A., Lanas Arbeloa A. (2015). Esophageal cancer: Risk factors, screening and endoscopic treatment in Western and Eastern countries. World J. Gastroenterol..

[55] Hur C., Miller M., Kong C.Y., Dowling E.C., Nattinger K.J., Dunn M., Feuer E.J. (2013). Trends in esophageal adenocarcinoma incidence and mortality. Cancer.

[56] Fiocca R., Mastracci L., Lugaresi M., Grillo F., D’Errico A., Malvi D., Spaggiari P., Tomezzoli A., Albarello L., Ristimaki A. (2021). The Prognostic Impact of Histology in Esophageal and Esophago-Gastric Junction Adenocarcinoma. Cancers.

[57] Angerilli V., Parente P., Campora M., Ugolini C., Battista S., Cassoni P., Gambella A., Cavallin F., De Lisi G., Vanoli A. (2023). HER2-low in gastro-oesophageal adenocarcinoma: A real-world pathological perspective. J. Clin. Pathol..

[58] Bozzarelli I., Orsini A., Isidori F., Mastracci L., Malvi D., Lugaresi M., Fittipaldi S., Gozzellino L., Astolfi A., Rasanen J. (2024). miRNA-221 and miRNA-483-3p Dysregulation in Esophageal Adenocarcinoma. Cancers.

[59] Lauren P. (1965). The Two Histological Main Types of Gastric Carcinoma: Diffuse and So-Called Intestinal-Type Carcinoma. An Attempt at a Histo-Clinical Classification. Acta Pathol. Microbiol. Scand..

[60] Ming S.C. (1977). Gastric carcinoma. A pathobiological classification. Cancer.

[61] Mariette C., Carneiro F., Grabsch H.I., van der Post R.S., Allum W., de Manzoni G. (2019). Consensus on the pathological definition and classification of poorly cohesive gastric carcinoma. Gastric Cancer.

[62] Siewert J.R., Stein H.J. (1996). Carcinoma of the gastroesophageal junction—Classification, pathology and extent of resection. Dis. Esophagus.

[63] Siewert J.R., Stein H.J. (1998). Classification of adenocarcinoma of the oesophagogastric junction. Br. J. Surg..

[64] Pera M., Pera M. (2001). Recent changes in the epidemiology of esophageal cancer. Surg. Oncol..

[65] Pohl H., Wrobel K., Bojarski C., Voderholzer W., Sonnenberg A., Rosch T., Baumgart D.C. (2013). Risk factors in the development of esophageal adenocarcinoma. Am. J. Gastroenterol..

[66] Cancer Genome Atlas Research Network (2014). Comprehensive molecular characterization of gastric adenocarcinoma. Nature.

[67] Nakamura Y., Kawazoe A., Lordick F., Janjigian Y.Y., Shitara K. (2021). Biomarker-targeted therapies for advanced-stage gastric and gastro-oesophageal junction cancers: An emerging paradigm. Nat. Rev. Clin. Oncol..

[68] Isidori F., Bozzarelli I., Mastracci L., Malvi D., Lugaresi M., Molinari C., Soderstrom H., Rasanen J., D’Errico A., Fiocca R. (2020). Targeted Sequencing of Sorted Esophageal Adenocarcinoma Cells Unveils Known and Novel Mutations in the Separated Subpopulations. Clin. Transl. Gastroenterol..

[69] Hezova R., Kovarikova A., Srovnal J., Zemanova M., Harustiak T., Ehrmann J., Hajduch M., Svoboda M., Sachlova M., Slaby O. (2015). Diagnostic and prognostic potential of miR-21, miR-29c, miR-148 and miR-203 in adenocarcinoma and squamous cell carcinoma of esophagus. Diagn. Pathol..

[70] Jiang X.H., Liu Q., Fu M., Wang C.F., Zou R.H., Liu L., Wang M. (2024). Long-term outcomes of endoscopic submucosal dissection for early esophageal adenocarcinoma in the Eastern population: A comprehensive analysis. J. Gastrointest. Surg..

[71] Lee C.T., Chang C.Y., Lee Y.C., Tai C.M., Wang W.L., Tseng P.H., Hwang J.C., Hwang T.Z., Wang C.C., Lin J.T. (2010). Narrow-band imaging with magnifying endoscopy for the screening of esophageal cancer in patients with primary head and neck cancers. Endoscopy.

[72] Yang Q., Liu Z., Sun H., Jiao F., Zhang B., Chen J. (2023). A narrative review: Narrow-band imaging endoscopic classifications. Quant. Imaging Med. Surg..

[73] Mochizuki Y., Saito Y., Kobori A., Ban H., Shioya M., Nishimura T., Inatomi O., Bamba S., Tsujikawa T., Ishida M. (2012). Magnified endoscopy combined with narrow band imaging of minimal superficial esophageal neoplasia-indicators to differentiate intraepithelial neoplasias. J. Gastrointest. Cancer.

[74] Dobashi A., Goda K., Yoshimura N., Ohya T.R., Kato M., Sumiyama K., Matsushima M., Hirooka S., Ikegami M., Tajiri H. (2016). Simplified criteria for diagnosing superficial esophageal squamous neoplasms using Narrow Band Imaging magnifying endoscopy. World J. Gastroenterol..

