# Supplementary material for: Pre-Surgical Endoscopic Biopsies Are Representative of Esophageal and Esophago-Gastric Junction Adenocarcinoma Histologic Classes and Survival Risk
Source: Cancers (Basel). 2024 Dec 2;16(23):4045. doi: 10.3390/cancers16234045 (PMC11640587; doi:10.3390/cancers16234045)
Supplement: Supplementary file 1 [file cancers-16-04045-s001.zip › cancers-3293287-supplementary.pdf]

Pre-Surgical Endoscopic Biopsies Are Representative of Esophageal and Esophago-Gastric Junction Adenocarcinoma Histologic Classes and Survival Risk

Alessandro Gambella, Roberto Fiocca, Marialuisa Lugaresi, Antonietta D'Errico, Deborah Malvi, Paola Spaggiari, Anna Tomezzoli, Luca Albarello, Ari Ristimäki, Luca Bottiglieri, Elena Bonora, Kausilia K. Krishnadath, Gian Domenico Raulli, Riccardo Rosati, Uberto Fumagalli Romario, Giovanni De Manzoni, Jari Räsänen, Sandro Mattioli, Federica Grillo, Luca Mastracci on behalf of the EACSGE Study Group

Supplementary Materials

Index

Item	Page
Supplementary Methods	2
Table S1. Clinico-pathological features of our series	3-4
Table S2. Details of the components of cases with mixed histopathologic subclass	5

Supplementary Methods

Inclusion criteria were: age >18 years; histological diagnosis of adenocarcinoma; availability of endoscopy/barium swallow reports for Siewert's classification; availability of the original pathology report, stained slides, and/or paraffin blocks from the surgical specimen; post-surgical follow-up data, including overall (OS) and cancer-specific (CSS) and disease-free (DFS) survival.

Exclusion criteria were pT1a/N0/M0 cases according to the AJCC 8th edition [26] and the following rare histotypes: adenosquamous, neuroendocrine carcinomas, or mixed neuroendocrine–non-neuroendocrine carcinomas (demonstrated by immunohistochemical expression of neuroendocrine markers). No patient treated with neoadjuvant therapy was included to ensure that tumor morphology was free from treatment-induced factors (e.g., fibrosis, loss of architectural features, post-treatment mucinous areas).

Table S1. Clinico-pathological features of our series.

Characteristic		Cases (n=106)
Clinical		
**Age, median (IQR)**		69.5 (59-76)
**Sex, male (%)**		84 (79.2%)
**Siewert type (%)**		
	**I**	33 (31.1%)
	**II**	54 (50.9%)
	**III**	19 (17.9%)
Histopathological		
**Surgical margins, free from disease (%)**		98 (92.4%)
**Lymph node**		
	**Resected, median (IQR)**	27 (19-35)
	**Involved, median (IQR)**	3 (1-8)
	**Ratio involved/resected, median (IQR)**	0.14 (0.02-0.35)
**pT**		
	**1**	1 (0.9)
	**2**	26 (24.5)
	**3**	73 (68.9)
	**4**	6 (5.7)
**pN**		
	**0**	25 (23.6)
	**1**	34 (32.1)
	**2**	22 (20.7)
	**3**	25 (23.6)
**pM**		
	**0**	93 (87.7)
	**1**	8 (7.6)
	**X**	5 (4.7)
**Vascular invasion**		
	**None**	49 (46.2%)
	**Evident**	57 (53.8%)
**Perineural invasion**		
	**None**	59 (55.7%)
	**Evident**	47 (44.3%)
**Stage (pathological)**		
	**1a**	0 (0%)
	**1b**	3 (2.8%)
	**2a**	10 (9.4%)
	**2b**	13 (12.3%)
	**3a**	11 (10.4%)
	**3b**	45 (42.5%)
	**4a**	22 (20.7%)
	**4b**	2 (1.9%)
**Lauren classification**		
	**Diffuse**	6 (5.7%)
	**Intestinal**	91 (85.8%)
	**Unclassified**	9 (8.5%)
**Ming classification**		
	**Expanding**	38 (35.8%)

**Infiltrative**	68 (64.2%)
**WHO grading**	
**G1**	43 (40.6%)
**G2**	30 (28.3%)
**G3**	33 (31.1%)
**Percentage of Non-Cohesive Components**	
**None**	80 (75.5%)
**1-10%**	7 (%)
**10-20%**	4 (%)
**20-30%**	2 (%)
**30-40%**	1 (%)
**40-50%**	1 (%)
**50-60%**	1 (%)
**60-70%**	0 (%)
**70-80%**	3 (%)
**80-90%**	3 (%)
**>90%**	4 (%)
**Percentage of Signet Ring Cells**	
**None**	84 (79.3%)
**1-10%**	9 (8.5%)
**10-20%**	8 (7.6%)
**20-30%**	1 (0.9%)
**30-40%**	0 (0%)
**40-50%**	3 (2.8%)
**50-60%**	0 (0%)
**60-70%**	0 (0%)
**70-80%**	0 (0%)
**80-90%**	1 (0.9%)
**>90%**	0 (0%)
Survival	
**Follow-up months, median (IQR)**	15 (6-36)
**OS**	
**Death**	43 (57.3%)
**Alive**	32 (42.7%)
**CSS**	
**Yes**	34 (45.3%)
**No**	41 (54.7%)
**Recurrence**	
**No**	37 (48.7%)
**Yes**	39 (51.3%)

Table S2. Details of the components of cases with mixed histopathologic subclass.

Case	Principal component	Secondary component
#1	PD-GAC	DAC
#2	PD-GAC	DAC
#3	PD-GAC	DAC
#4	PD-GAC	DAC
#5	PD-GAC	DAC
#6	PD-GAC	DAC
#7	PD-GAC	DAC
#8	WD-GAC	DAC
#9	MMC	DAC
